# Long‐term outcomes of combined intravitreal methotrexate and systemic high‐dose methotrexate therapy in vitreoretinal lymphoma

**DOI:** 10.1002/cam4.5609

**Published:** 2023-01-05

**Authors:** Chieh‐Lung Cheng, Po‐Ting Yeh, Wei‐Quan Fang, Wei‐Li Ma, Hsin‐An Hou, Cheng‐Hong Tsai, Chang‐Ping Lin, Hwei‐Fang Tien

**Affiliations:** ^1^ Division of Hematology, Department of Internal Medicine, National Taiwan University Hospital, College of Medicine National Taiwan University Taipei Taiwan; ^2^ Department of Ophthalmology, National Taiwan University Hospital, College of Medicine National Taiwan University Taipei Taiwan; ^3^ Division of New Drug Center for Drug Evaluation Taipei Taiwan; ^4^ Department of Oncology, National Taiwan University Hospital, College of Medicine National Taiwan University Taipei Taiwan

**Keywords:** combination treatment, methotrexate, relapse, vitreoretinal lymphoma

## Abstract

**Objective:**

The optimal treatment for vitreoretinal lymphoma (VRL) remains a challenge, as central nervous system (CNS) relapse occurs frequently, leading to the worst impact on survival. We previously proposed combined intravitreal methotrexate and systemic high‐dose methotrexate therapy for this disease. This study aimed to report the long‐term outcomes of patients with VRL using this combination treatment.

**Methods:**

We conducted a retrospective cohort study on patients with VRL at a tertiary referral center between 2003 and 2018.

**Results:**

Thirty‐two patients were included, of whom 23 had primary VRL (PVRL) and nine had concurrent intraocular and CNS diseases. The treatment was well tolerated. Twenty‐six (81.3%) patients achieved complete response (CR). After a median follow‐up time of 103.5 months, the 5‐year survival rate was 73.3%, whereas the 5‐year progression‐free survival (PFS) rate was 29.9%. Twenty‐four (75%) patients relapsed, including 12 with isolated intraocular relapses at first relapse and a total of 17 with CNS/systemic relapses. The development of CNS/systemic relapse negatively affected survival, but intraocular relapse did not. The median CNS/systemic PFS was 69.5 months, but the risk of CNS/systemic relapse increased steadily with a cumulative incidence rate at 2, 5, and 10 years being 22.6%, 44.2%, and 65%, respectively. Multivariate analysis identified concurrent CNS disease at diagnosis as the only poor‐risk factor for CNS/systemic relapse.

**Conclusions:**

This study confirms good efficacy and acceptable toxicities of the combination approach. However, incorporation of further intensive consolidation strategies into the treatment protocol to effectively prevent subsequent CNS/systemic relapse deserves to be considered.

## Graphical abstract

Our study showed the long‐term outcomes regarding the combined intravitreal MTX and systemic HDMTX therapy in patients with vitreoretinal lymphoma (VRL). This combination approach induced a high survival rate with acceptable toxicities. Nevertheless, more than half of patients experienced CNS/systemic relapse, accounting for the main cause of death. Incorporation of more intensive consolidation strategies into the treatment protocol deserves to be considered, especially for patients with concurrent CNS disease at diagnosis.

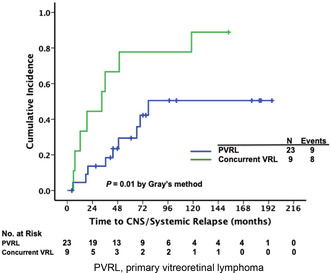

## 1 INTRODUCTION

Vitreoretinal lymphoma (VRL) is the most common intraocular lymphoma worldwide.^1^ At initial diagnosis, VRL may be restricted to the intraocular compartment without brain or cerebrospinal fluid (CSF) involvement, called primary VRL (PVRL), or have concurrent central nervous system (CNS) diseases. Like primary CNS lymphoma, the majority of VRL cases are high‐grade non‐Hodgkin lymphoma, belonging to the activated B‐cell subtype of diffuse large B‐cell lymphoma.^2^ However, few cases of T cell or NK cell origin had been reported.^3^, ^4^, ^5^ The median age at VRL diagnosis is in the fifth–sixth decades of life.^6^, ^7^ It seems that VRL has no racial predilection.^1^ Additionally, growing evidence shows that women are more commonly affected than men.^5^, ^8^, ^9^, ^10^

Patients with VRL commonly have bilateral ocular involvements.^1^, ^11^, ^12^ Blurred vision and floaters are the most common ophthalmologic symptoms. Notably, these nonspecific clinical manifestations mimic posterior uveitis, letting VRL been termed as a masquerade syndrome.^7^ The golden standard for the diagnosis of VRL is the cytologic identification of lymphoma cells in the vitrectomy specimens.^13^ Flow cytometry, measurements of interleukin levels,^14^ detection of immunoglobulin heavy chain or T‐cell receptor gene rearrangements,^15^, ^16^ more recently novel molecular biomarker tests of vitreous samples,^17^, ^18^, ^19^ as well as the multimodal retinal imaging in the ophthalmic field^6^, ^20^ all contribute to the improvement of diagnostic yield.

VRL usually has a good response to initial treatment. Nevertheless, subsequent local or CNS relapse frequently occurs. It has been reported that over half of patients with VRL will eventually develop CNS relapse or progression, which accounts for the most frequent cause of death.^7^, ^21^ Nowadays, several therapeutic strategies are available for VRL: local intraocular therapy including ocular radiotherapy or intravitreal (IVT) injection of methotrexate or rituximab; systemic high‐dose methotrexate (HDMTX)‐based chemotherapy with or without intrathecal (IT) therapy; or a combination of both. However, due to the rarity of the disease, the best frontline therapeutic strategy for VRL remains a matter of debate.^13^

Despite no standard therapy for VRL, the goal of treatment is to achieve complete response (CR) of intraocular disease for restoring the visual acuity and reduce the risk of subsequent CNS or systemic relapse. In 2003, we had adopted the therapy combining IVT methotrexate and systemic HDMTX with or without IT therapy in the management of all patients with newly diagnosed VRL at the National Taiwan University Hospital (NTUH).^22^ IVT methotrexate is used to quickly reach effective tumoricidal level to eliminate the intraocular disease, while systemic HDMTX with or without IT therapy is intended to eradicate the concurrent CNS disease or potentially microscopic CNS seeding to prevent the subsequent relapses. In this study, we expanded the patient number from 19 in the previous report to 32 and presented an update on the long‐term follow‐up outcomes of patients with VRL treated with this combination approach.

## 2 MATERIALS AND METHODS

### 2.1 Patient selection and clinical data collection

From April 2003 to December 2018, patients with newly diagnosed VRL without a concurrent human immunodeficiency virus infection at NTUH were considered for enrolment. For the diagnosis of VRL, all enrolled patients underwent diagnostic vitrectomy to obtain adequate vitreous samples for cytologic and flow cytometric analyses. VRL was diagnosed when large, abnormal lymphoid cells with high nuclear/cytoplasm ratio, pleomorphic nuclei, and basophilic cytoplasm were identified by a cytologic examination of the vitreous samples. The immunophenotype and monoclonality of the abnormal cells were determined by flow cytometry. Additionally, all patients underwent CSF analysis, brain magnetic resonance imaging (MRI), bone marrow biopsy, and whole‐body computed tomography (CT) with contrast or positron emission tomography/CT to clarify the disease extent. VRL with disease limiting to the intraocular compartment was defined as PVRL, whereas VRL with concomitant brain or CSF involvement was defined as concurrent VRL.

We performed a retrospective chart review to collect data on clinical features, treatment responses, relapse events, and outcomes. Treatment‐related toxicities were evaluated in accordance with the National Cancer Institute Common Terminology Criteria for Adverse Events Version 5.

### 2.2 Combination therapy

All enrolled patients had received combined IVT methotrexate and systemic HDMTX therapy as the frontline treatment. IVT injection of methotrexate was performed at a dose of 400 μg. For the dosage of HDMTX, it was administered at a target dose of 8 g/m^2^ over 6 h per cycle. Dose reduction of methotrexate to 6 g/m^2^ was acceptable, especially for older patients. The detailed treatment protocol of the combination approach has been described in our previous study.^22^ Patients with concomitant CSF involvement also received concurrent IT chemotherapy consisting of methotrexate, cytarabine, and hydrocortisone.

### 2.3 Treatment response evaluation

The assessment of therapeutic response was scheduled to perform after 1 month of treatment and was followed up at intervals of 3 months thereafter. CR is defined as the disappearance of lymphomatous infiltrates within the eye or CNS as determined by ophthalmologic evaluation, brain MRI scan, and CSF examination. Partial response (PR) is defined as more than 50% reduction of lymphomatous infiltrates. Progressive disease (PD) is defined as deterioration of previous ocular or CNS lesions, and stable disease is defined as less than a PR but is not PD. Relapsed disease means reappearance of any new lesion after achieving CR.

### 2.4 Statistical analysis

A chi‐square test or Fisher's exact test was used to compare categorical data. A Mann–Whitney *U* test was used to compare the medians of continuous variables. Overall survival (OS) was measured from the date of diagnosis to death from any cause or the date of the last follow‐up. Progression‐free survival (PFS) was measured from the date of diagnosis until the earliest occurring time point among the following: the end of the follow‐up period, the date of relapse (regardless of location), PD, or death from any cause. CNS/systemic PFS was defined as the time without CNS and/or systemic relapse. Cumulative incidence curves were determined for relapse events, and Gray's test was used to examine the significance of between‐group differences. The binary logistic regression analysis was performed to identify the independent risk factors associated with CNS/systemic relapse. The Kaplan–Meier method was used to calculate the curves of OS and PFS, and a log‐rank test was used to identify significant between‐group differences. Hazard ratios (HRs) and 95% confidence intervals (CIs) were estimated using univariate and multivariate Cox proportional hazard regression models to determine the independent risk factors associated with survival. A two‐sided *p* value <0.05 was considered statistically significant. All statistical analyses were performed using SPSS version 21 software (IBM).

## 3 RESULTS

### 3.1 Patient characteristics

A total of 32 patients were evaluated, with a median age at diagnosis being 63 years (range: 39–78 years) and 22 (68.8%) being women. Among them, 23 had PVRL and nine had concurrent VRL, and 18 had unilateral and 14 had bilateral ocular involvements. The clinical characteristics of patients with VRL are shown in Table 1. Furthermore, the data of cytologic and flow cytometric analyses of the vitreous fluid in three representative patients are presented in Figure S1. The median duration of symptoms before diagnosis was 6 months (range: 2–32 months). All patients presented with ocular complaints without associated neurologic symptoms or signs at diagnosis. The most common presenting symptoms were blurred vision in 26 (81.3%) and floaters in 15 (46.9%) patients. The disease laterality was not associated with concomitant CNS involvement.

**TABLE 1:** Clinical characteristics of 32 patients with VRL.

Patient demographics	*N* = 32
	Number of patients (%)
Median age at diagnosis (range)	63 (39–78)
Sex	
Male	10 (31.2)
Female	22 (68.8)
Clinical features	
Disease involvement	
Eye	23 (71.9)
Eye + CSF	5 (15.6)
Eye + brain	1 (3.1)
Eye + CSF + brain	3 (9.4)
Laterality	
Bilateral	14 (43.7)
Unilateral	18 (56.3)
Right	8 (44.4)
Left	10 (55.6)
Visual symptoms	
Blurred vision	26 (81.3)
Floaters	15 (46.9)
Lymphoma subtypes	
B cell	30 (93.8)
T cell	1 (3.1)
NK cell	1 (3.1)
High‐dose MTX	
8 g/m^2^	23 (71.9)
6 g/m^2^	9 (28.1)
Median number of MTX injections (range)	10 (3–12)
Treatment response to induction therapy	
Complete response	26 (81.3)
Progressive disease	1 (3.1)
Stable disease	5 (15.6)
Autologous stem cell transplantation	5 (15.6)
WBRT	
Second‐line therapy	4 (12.5)
At relapse	7 (21.9)

Abbreviations: CSF, cerebrospinal fluid; MTX, methotrexate; VRL, vitreoretinal lymphoma; WBRT, whole‐brain radiotherapy.

### 3.2 Treatment

The median number of IVT methotrexate injections was 13 (range: 5–18), and the median number of HDMTX injections was 10 (range: 3–12). Regarding systemic HDMTX, 23 (71.9%) patients received systemic HDMTX at a dose of 8 g/m^2^ per cycle. The remaining nine patients received 6 g/m^2^ HDMTX per injection.

### 3.3 Treatment response

The treatment responses to the combination therapy are presented in Table 1. Twenty‐six (81.3%) patients achieved CR, 87% in the PVRL subgroup, and 66.7% in the concurrent VRL subgroup (*p* = 0.314). Intriguingly, the six nonresponder patients all achieved CR after the salvage therapies.

### 3.4 Relapse events

During the median follow‐up period of 103.5 months (range: 4.4–195.2 months), 24 (75%) patients experienced relapse events. The median time to first relapse was 20.3 months (95% CI, 12.9–27.7 months). Of these 24 patients, 11 had isolated intraocular relapse, 12 had CNS/systemic relapse, and one had simultaneous intraocular and CNS involvement at the first relapse.

The clinical characteristics and treatment outcomes of the 11 patients with isolated intraocular involvement at first relapse are displayed in Table S1. All 11 patients received more frequent IVT chemotherapy as the salvage therapy, and one of them also received consolidated whole‐brain radiotherapy (WBRT). They all achieved a second CR. Afterward, four patients had continuous CR, three had subsequent intraocular relapse alone, and four developed subsequent CNS/systemic relapse. The three patients with serial intraocular relapse alone were all alive without disease after further treatments. Nevertheless, two of the four patients with subsequent CNS/systemic relapse died of disease (Cases 14 and 19, Table S1).

The only one patient with simultaneous intraocular and CNS involvement at first relapse received carmustine‐based systemic chemotherapy in combination with IVT chemotherapy as the salvage therapy (Case 6, Table 2). Nevertheless, the disease rapidly progressed, and the patient died of disease in 2 months.

**TABLE 2:** Clinical features and treatment outcomes of the 17 patients with CNS/systemic relapse

Case	Age at relapse	Sex	First relapse			Second relapse			Third relapse			Fourth relapse		
			Site of relapse	Therapy	Outcomes	Site of relapse	Therapy	Outcomes	Site of relapse	Therapy	Outcomes	Site of relapse	Therapy	Outcomes
2^a^	54	F	BM + Lung	CHOP + ASCT	CR in 4.5 m	CNS	BOMES + WBRT	CR in 3.5 m	BM	CHOP	Died in 1.5 m			
3	53	M	CNS	WBRT	CR in 27.5 m	CNS	BOMES	CR in 9.5 m	CNS	BAES	Died in 5 m			
6	55	F	CNS + Left eye	carmustine/cytarabine + IVT chemotherapy	Died in 2 m									
12	73	F	CNS	BMS	CR in 7 m	Liver	R‐ESHAP	CR in 34.5 m	Skin	R‐CHOP	CR in 1.5 m	Sinonasal	BR + Lenalidomide	Died in 4.5 m
14^a^	40	F	Left eye	IVT chemotherapy	CR in 4.5 m	CNS	BAS + WBRT + BR	Died in 10 m						
15^a^	66	F	CNS	R‐HDMTX + WBRT	CR in 8.5 m									
16	75	F	CNS	R‐HDMTX	CR in 12.5 m	CNS	Lenalidomide + local radiotherapy	CR in 3 m	CNS	Rituximab + Lenalidomide	Died in 5 m			
17^a^	47	M	CNS	R‐CYVE + ASCT	CR in 3.5 m	CNS	HDMTX	Died in 2.5 m						
18	55	F	Left eye	IVT chemotherapy	CR in 17 m	Extensively systemic + CNS	R‐CHOP/R‐CYVE + ASCT	CR in 60 m						
19	75	M	Left eye	IVT chemotherapy	CR in 24.5 m	Right eye	IVT chemotherapy	CR in 7 m	Right eye	IVT chemotherapy	CR in 4.5 m	Lymph nodes	R‐miniCHOP	Died in 2.5 m
20	79	F	Left eye	IVT chemotherapy	CR in 20 m	CNS	R‐HDMTX + WBRT	CR in 46 m						
22	64	F	CNS + Paraspinal	R‐ESHAP + ASCT	CR in 51 m									
23^a^	52	M	CNS	CYVE + ASCT	CR in 42 m									
26^a^	63	M	CNS	WBRT	Died in 2 m									
28^a^	78	M	CNS	WBRT	CR in 30 m	CNS	Rituximab + Lenalidomide	Died in 11 m						
31	73	F	BM	GEPS	Died in 1 m									
32^a^	55	F	Lymph nodes	R‐BOMES/R‐IVAC	CR in 20 m									

Abbreviations: ASCT, autologous stem cell transplantation; BAS, carmustine, cytarabine, and steroid; BM, bone marrow; BMS, carmustine, methotrexate, and steroid; BR, bendamustine and rituximab; CHOP, cyclophosphamide, doxorubicin, oncovin, and prednisolone; CNS, central nervous system; CR; complete response, BOMES, carmustine, oncovin, methotrexate, etoposide, and steroid; CYVE, cytarabine and etoposide; F, female; GEPS, gemcitabine, etoposide, cisplatin, and steroid; HDMTX, high‐dose methotrexate; IVAC, ifosfamide/mesna, etoposide, and cytarabine; IVT, intravitreal; M, male; R, rituximab; ESHAP, etoposide, cytarabine, cisplatin, and steroid; WBRT, whole‐brain radiotherapy; BAES, carmustine, cytarabine, etoposide, and steroid.

^a^ Patients with concurrent vitreoretinal lymphoma at diagnosis.

Overall, intraocular relapse was observed in 12 (37.5%) patients. The median time to intraocular relapse was 20.3 months (95% CI, 12.2 to 28.4 months). The cumulative incidence rate (CIR) of intraocular relapse at 2 and 5 years were respectively 20.6% and 42%, reaching a plateau at 33.6 months (Figure S2). The incidence of intraocular relapse did not differ between PVRL and concurrent VRL subgroups (*p* = 0.422).

**FIGURE 1:**
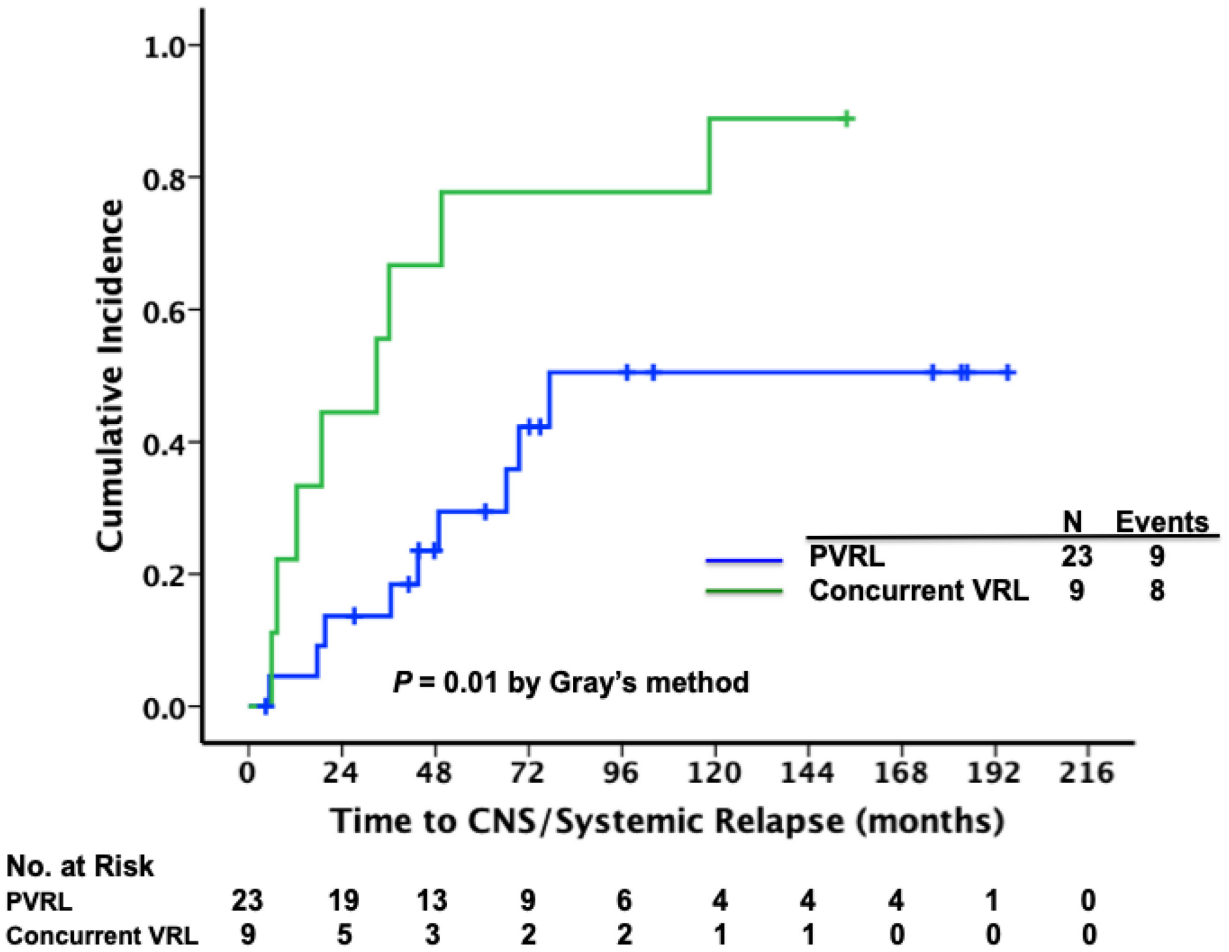
Cumulative incidence of central nervous system (CNS)/systemic relapse in patients with vitreoretinal lymphoma (VRL), stratified by the presence or absence of concurrent CNS disease at diagnosis. A higher cumulative incidence rate (CIR) of CNS/systemic relapse was observed in patients with concurrent intraocular and CNS disease (concurrent VRL) than in patients with primary VRL (PVRL). The CIR of CNS/systemic relapse at 2, 5, and 10 years were respectively 44.4%, 77.8%, and 88.9% in patients with concurrent VRL and 13.6%, 29.4%, and 50.5% in patients with PVRL.

Throughout the follow‐up period, 17 (53.1%) patients experienced CNS/systemic relapse. The median time to CNS/systemic relapse was 36.1 months (95% CI, 13.3 to 58.9 months). The CIR of CNS/systemic relapse at 2, 5, and 10 years were 22.6%, 44.2%, and 65%, respectively (Figure S3). Furthermore, CNS/systemic relapse was observed in nine (39.1%) patients with PVRL and eight (88.9%) patients with concurrent VRL, respectively. Compared with the patients with PVRL, those with concurrent VRL at diagnosis had a higher CIR of CNS/systemic relapse (2, 5, and 10‐year CIRs, 44.4%, 77.8%, and 88.9% in patients with concurrent VRL versus 13.6%, 29.4%, and 50.5% in those with PVRL, *p* = 0.01, Figure 1).

The clinical features and treatment outcomes of the 17 patients with CNS/systemic relapse are summarized in Table 2. The therapeutic strategies for CNS/systemic relapse were determined as the discretion of the primary ‐care hematologists. Eleven of 17 patients with CNS/systemic relapse eventually died of disease.

### 3.5 Survival

After a median follow‐up time of 103.5 months, the median OS had not been reached. OS rate at 5 and 10 years was 73.3% and 58.9%, respectively (Figure S4A). The median PFS was 20.3 months (95% CI, 11.8 to 28.8 months), and the 2 and 5‐year cumulative PFS rates were 50% and 29.9%, respectively (Figure S4B). Additionally, the median CNS/systemic PFS was 69.5 months (95% CI, 31.5 to 107.5 months, Figure S5). Twenty patients remained alive at the time of last follow‐up, while 12 patients died—11 from recurrent disease and the remaining one from infection.

**FIGURE 2:**
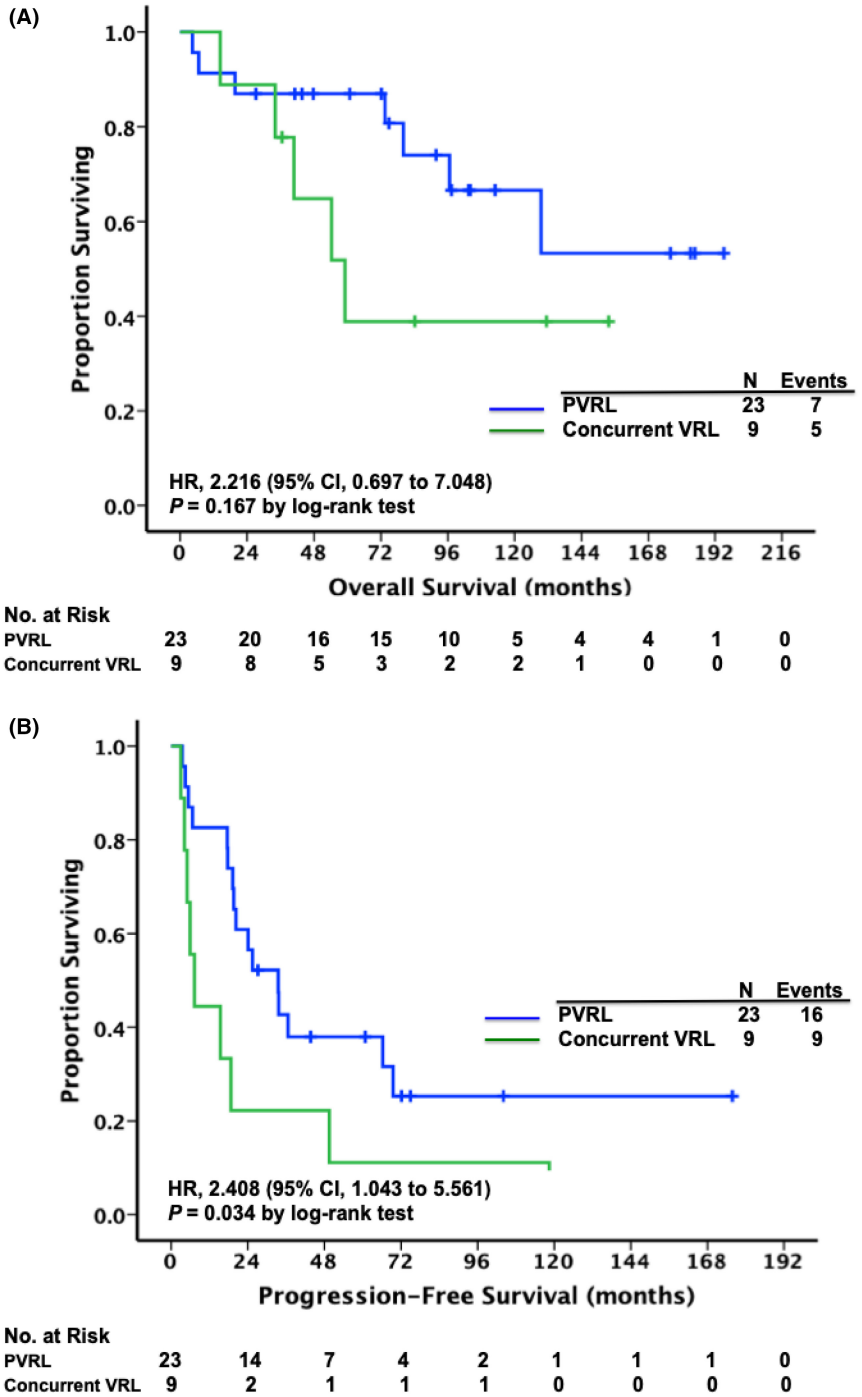
Kaplan–Meier survival curves in patients with vitreoretinal lymphoma (VRL), stratified by the presence or absence of concurrent central nervous system (CNS) disease at diagnosis. Patients with concurrent intraocular and CNS disease (concurrent VRL) had a trend of poorer overall survival (A) and significantly shorter progression‐free survival (B) than patients with primary VRL (PVRL).

Age, sex, and disease laterality had no prognostic implication in our cohort. Compared with the patients with PVRL, those with concurrent VRL had a trend of poorer OS (median OS, 59.1 months vs. not reached, log‐rank *p* = 0.167, Figure 2A) and a significantly shorter PFS (median PFS, 7.3 vs. 33.5 months, log‐rank *p* = 0.034, Figure 2B). Additionally, a shorter CNS/systemic PFS was observed in patients with concurrent VRL than in those with PVRL (median CNS/systemic PFS, 30.7 vs. 77.3 months, log‐rank *p* = 0.012, Figure S6). Intraocular relapse had no impact on OS. In contrast, multivariate analysis identified CNS/systemic relapse as an independent unfavorable factor for OS (HR: 11.315; 95% CI: 1.335–95.892; *p* = 0.026, Table 3). The 2 and 5‐years OS rates post‐CNS/systemic relapse were 58.2% and 34.5%, respectively.

**TABLE 3:** Univariate and multivariate analyses of risk factors for overall survival in patients with VRL

Variables	Univariate analysis			Multivariate analysis		
	HR	95% CI	*p* value	HR	95% CI	*p* value
Age >60 years	0.967	0.304–3.076	0.955	1.589	0.405–6.23	0.506
Male sex	0.601	0.189–1.908	0.387	0.615	0.1722.204	0.456
Bilateral	1.067	0.337–3.371	0.912	1.045	0.279–3.919	0.948
Concurrent CNS disease	2.216	0.697–7.048	0.178	1.319	0.284–6.115	0.724
Ocular relapse	0.405	0.108–1.514	0.179	0.778	0.17–3.569	0.747
CNS/systemic relapse	11.85	1.512–92.852	0.019	11.315	1.335–95.892	0.026

Abbreviations: CI, confidence interval; CNS, central nervous system; HR, hazard ratio; VRL, vitreoretinal lymphoma.

### 3.6 Risk factors associated with CNS/systemic relapse

Several clinical features, including age, sex, disease laterality, and concomitant CNS involvement at diagnosis, were examined to explore the factors predictive of CNS or systemic relapse. By multivariate binary logistic regression analysis, we identified that concomitant CNS involvement at diagnosis is the only risk factor significantly associated with the development of CNS and/or systemic relapse (Table 4).

**TABLE 4:** Binary logistic regression analysis of clinical factors associated with CNS/systemic relapse in patients with VRL.

Predictors	Univariate analysis			Multivariate analysis		
	OR	95% CI	*p* value	OR	95% CI	*p* value
Age >60 years	0.594	0.227–1.553	0.288	0.989	0.317–3.087	0.984
Male sex	0.785	0.289–2.127	0.634	1.025	0.35 –2.997	0.964
Bilateral	0.588	0.207–1.67	0.319	0.423	0.126–1.422	0.164
Concurrent CNS disease	3.237	1.235–8.486	0.017	4.005	1.213–13.224	0.023

Abbreviations: CI, confidence interval; CNS, central nervous system; OR, odds ratio; VRL, vitreoretinal lymphoma.

### 3.7 Treatment‐related adverse effects

The most common adverse effect of IVT methotrexate was superficial punctate keratitis, which occurred in 21 (65.6%) patients. Dry eye and sterile endophthalmitis were occurred in five (15.6%) and two (6.3%) patients, respectively. Only one (3.1%) patient developed maculopathy, and none had a vitreous hemorrhage.

Concerning the hematologic toxicities, grade 3/4 neutropenia was observed in four (12.5%) and grade 3/4 thrombocytopenia in three (9.4%) patients. No grade of anemia was recorded. Two out of 32 patients also developed grade 4 febrile neutropenia. Intriguingly, all but one (31/32, 96.9%) patients developed hepatotoxicity, of which 15 was grade 1, six was grade 2, and 10 was grade 3 toxicity. Nonetheless, the hepatotoxicity was reversible, and no late sequelae were reported. Seven (21.9%) patients developed HDMTX‐related nephrotoxicity, among whom one had grade 1 and six had grade 2 toxicity. Notably, the renal function was completely recovered in six of the seven patients during the posttreatment follow‐up period. Overall, there was no toxic death or premature discontinuation of HDMTX due to toxicity.

## 4 DISCUSSION

VRL is a rare intraocular malignancy with a slight female predominance. Our study also showed that approximately 70% of patients with VRL were female. In contrast to previous studies that VRL are mainly bilateral, more than half of patients in our study had unilateral involvement at diagnosis. Our finding might underestimate the true value because VRL is often asymmetrical and initially may present as being unilateral.^23^ VRL commonly masquerades as posterior uveitis, which is responsible for its diagnostic delay. In our study, the median time from the onset of clinical symptoms to the definite diagnosis of VRL was 6 months, comparably with other reported data.^12^, ^24^, ^25^

We had previously reported the efficacy of combined IVT methotrexate and systemic HDMTX treatment in 19 patients with VRL.^22^ During a median follow‐up period of 40 months, the 5‐year survival rate was 55.8%. CNS/systemic relapses arose in six (31.6%) patients. In this extended and updated study with more patients enrolled, the overall survival remained high (5‐year survival rate of 73.3%), supporting the survival benefit regarding this combination approach. Nevertheless, a higher proportion of patients (17/32, 53.1%) experienced CNS/systemic relapse during a longer follow‐up period (9/23 [39.1%] among PVRL, 8/9 [88.9%] among concurrent VRL). Furthermore, the CIR of CNS/systemic relapse increased steadily over time, especially for the concurrent VRL subgroup. Intriguingly, late CNS relapse occurred in one patient beyond 10 years from diagnosis, underlying the inherent risk of CNS relapse in VRL. All these results suggest that the present combination approach could not effectively reduce the occurrence of subsequent CNS/systemic relapse, even if systemic HDMTX was given for a relatively high number of cycles (median 10 cycles).

The effectiveness of combined IVT methotrexate and systemic HDMTX therapy in VRL had been addressed. Akiyma et al. prospectively investigated 10 patients with PVRL treated with IVT methotrexate till CR of ocular lesions followed by five cycles of HDMTX (3.5 g/m^2^).^26^ Even though all patients achieved CR, a significant portion (40%) of them experienced CNS relapse. Hashida et al. retrospectively explored the association between CNS prophylactic treatments and CNS relapse in 26 patients with PVRL and reported that the time to CNS progression was significantly prolonged with the combined approach versus IVT therapy alone.^27^ However, four of six (66.7%) patients using systemic HDMTX as the preventive therapy eventually developed CNS relapse. More recently, a retrospective study of 59 patients with PVRL showed a high CR rate and a long median brain‐free survival after the HDMTX‐based polychemotherapies.^10^ Nevertheless, a total of 25 (42.4%) patients ultimately developed brain/systemic relapses. These studies further support our perspective that systemic HDMTX therapy can't effectively prevent the onset of CNS/systemic relapse, even if it can considerably prolong the time to subsequent CNS/systemic involvement.

Previous studies had demonstrated heterogeneous outcomes while using different forms of combination therapy in VRL treatment. From a small prospective study enrolling 17 patients, Kaburaki et al. reported a relatively low 4‐year CIR of CNS progression (14.3%) after a conventional PCNSL‐like treatment consisting of rituximab, HDMTX, procarbazine, and vincristine (R‐MPV) with IVT methotrexate as the induction therapy followed by consolidative reduced‐dose WBRT and high‐dose cytarabine (HDAC).^28^ In addition, de la Fuente et al. reported the outcomes of combined modality comprised of bilateral ocular radiotherapy followed by systemic HDMTX‐based chemotherapy in 12 patients with PVRL.^25^ With a median follow‐up time of 68 months, a low 5‐year CIR of CNS relapse (37.5%) was observed but at the expense of radiation retinopathy, cataract, and optic atrophy. By contrast, Cheah et al demonstrated a high 4‐year CIR of CNS progression (58%) in a retrospective single‐center study consisting of 11 patients with PVRL treated with R‐MPV followed by binocular radiotherapy and HDAC.^29^ Whether the incorporation of ocular radiotherapy, WBRT, or intensive chemotherapy into the combination strategy results in lower incidence of subsequent CNS/systemic relapse needs to be further investigated.

Despite similar CR rates after the combination approach, concurrent VRL was associated with a significantly higher CIR of CNS/systemic relapse than PVRL. Moreover, all but one patient with concurrent VRL developed CNS/systemic relapse, indicating that systemic HDMTX monotherapy adopted in this study is not enough to eliminate the occult CNS or systemic diseases in this subgroup of patients. Thus, the PCNSL‐like treatment^30^, ^31^ is highly recommended for patients with concurrent VRL, even if only asymptomatic meningeal involvement is detected, to reduce the risk of subsequent CNS/systemic recurrence.

Systemic relapse in VRL is rarely discussed in the literature. Among our cohort, seven patients (five [21.7%] with PVRL and two [22.2%] with concurrent VRL) experienced systemic relapse within nearly 9 years of follow‐up, and four of them eventually died of the disease. Of note, one patient developed systemic relapse <6 months from diagnosis, implying the presence of occult systemic lesions that were resistant to the frontline HDMTX monotherapy. Whether the addition of other chemotherapeutic regimens into HDMTX is more effective than HDMTX monotherapy to eradicate the occult systemic lesions in VRL requires further explorations.

More recently, increased insight into the pathophysiology of VRL has contributed to the development of several novel agents for this rare malignancy, especially for the Bruton's tyrosine kinase inhibitors (BTKi).^32^, ^33^ Guan et al. also demonstrated a high disease control rate (90%) after 1 month of BTKi monotherapy in a prospective, single‐center, phase 2 study enrolling 10 patients with relapsed or newly diagnosed VRL, paving the way for a paradigm change in the choices of VRL treatment.^34^

The main limitation of this study is the inherent biases due to the retrospective nature. Additionally, only one type of systemic treatment was performed, so the difference in the comparison between HDMTX‐based polychemotherapies and HDMTX monotherapy as the frontline regimen in VRL treatment couldn't be known. Furthermore, we did not routinely examine the levels of interleukins or novel biomarkers in vitreous or aqueous samples at diagnosis and at the end of treatment, which might influence the ocular assessments in our study. Nevertheless, the treatment adopted in our study is relatively homogeneous and the length of follow‐up was sufficient to draw conclusions. Our study truly showed the real‐world data regarding the use of combined IVT methotrexate and systemic HDMTX monotherapy in a relatively large cohort of Asian patients with VRL.

To sum up, we demonstrated the long‐term outcomes regarding the combination approach of IVT methotrexate and systemic HDMTX monotherapy as the frontline therapy for patients with VRL. This combination approach was well tolerated with acceptable toxic profiles. Moreover, it induced a high CR and survival rates as well as a long median CNS/systemic PFS. During an extended follow‐up period of nearly 9 years, nonetheless, more than half of the patients experienced CNS/systemic relapse, which represented the main cause of death in these patients. Intriguingly, constant pattern of CNS/systemic relapse was observed, indicating the suboptimal efficacy of this combination approach to prevent subsequent CNS/systemic progression. Incorporation of more intensive consolidation strategies into the treatment of VRL deserves to be considered, especially for patients with concurrent CNS disease at diagnosis.

## AUTHOR CONTRIBUTIONS

**Chieh‐Lung Cheng:** Conceptualization (lead); data curation (lead); formal analysis (lead); investigation (lead); methodology (lead); resources (equal); writing – original draft (lead). **Po‐Ting Yeh:** Data curation (supporting); investigation (equal); resources (equal); writing – original draft (supporting). **Wei‐Quan Fang:** Formal analysis (equal); methodology (equal); software (equal). **Wei‐Li Ma:** Data curation (equal); investigation (equal); resources (equal). **Hsin‐An Hou:** Data curation (equal); investigation (equal); resources (equal). **Cheng‐Hong Tsai:** Data curation (equal); investigation (equal); resources (equal). **Chang‐Ping Lin:** Data curation (equal); investigation (equal); resources (equal). **Hwei‐Fang Tien:** Conceptualization (lead); data curation (equal); investigation (equal); resources (equal); supervision (lead); writing – review and editing (lead).

## FUNDING INFORMATION

The authors declare no funding.

## CONFLICTS OF INTEREST

The authors reported no potential conflicts of interest.

## ETHICS STATEMENT

This study was approved by the Research Ethics Committee of National Taiwan University Hospital and was performed in compliance with the Declaration of Helsinki (IRB No. 202005129RINC).

## Supporting information


Appendix S1

Appendix S2

## Data Availability

The data that support the findings of this study are available from the corresponding author upon reasonable request.
