# Supplementary material for: Long‐term outcomes of combined intravitreal methotrexate and systemic high‐dose methotrexate therapy in vitreoretinal lymphoma
Source: Cancer Med. 2023 Jan 5;12(7):8102–11. doi: 10.1002/cam4.5609 (PMC10134364; doi:10.1002/cam4.5609)
Supplement: Supplementary file 1 — Appendix S1 [file CAM4-12-8102-s001.docx]

**Supporting information**

Figure. S1 **The data of cytologic and flow cytometric analyses of the vitreous fluid in three representative patients with vitreoretinal lymphoma.** (A) The lymphoma cells are characterized by high nuclear/cytoplasm (N/C) ratio, pleomorphic nuclei, and basophilic cytoplasm containing numerous small vacuoles. These cells express CD19/surface lambda light chain restriction, compatible with B-cell lymphoma (Liu’s stain; original magnification, ×1000). (B) The large and bizarre cells with cytoplasmic granules express CD56 and CD2 but do not express CD3, CD19 or surface light chain (red dots), compatible with NK-cell lymphoma (Liu’s stain; original magnification, ×1000). (C) The lymphoma cells are characterized by high N/C ratio, pleomorphic nuclei, and prominent nucleoli. Almost all these cells express CD3 and CD5; compatible with T-cell lymphoma (Liu’s stain; original magnification, ×1000).

Figure. S2 **Cumulative incidence of intraocular relapse in total cohort.**

Figure. S3 **Cumulative incidence of central nervous system (CNS)/systemic relapse in total cohort.**

Figure. S4 **Kaplan–Meier curves for overall survival (A) and progression-free survival (B) in total cohort.**

Figure. S5 **Kaplan–Meier curve for central nervous system/systemic progression-free survival in all patients with vitreoretinal lymphoma (VRL).**

Figure. S6 **Kaplan–Meier curves for central nervous system/systemic progression-free survival in all patients with vitreoretinal lymphoma (VRL), stratified by the presence or absence of concurrent central nervous system disease at diagnosis.**

Figure. S1

| 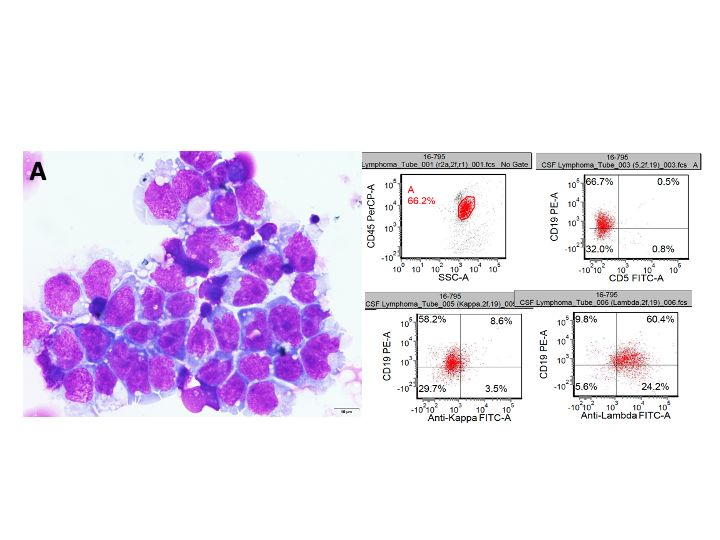 |
| --- |
| 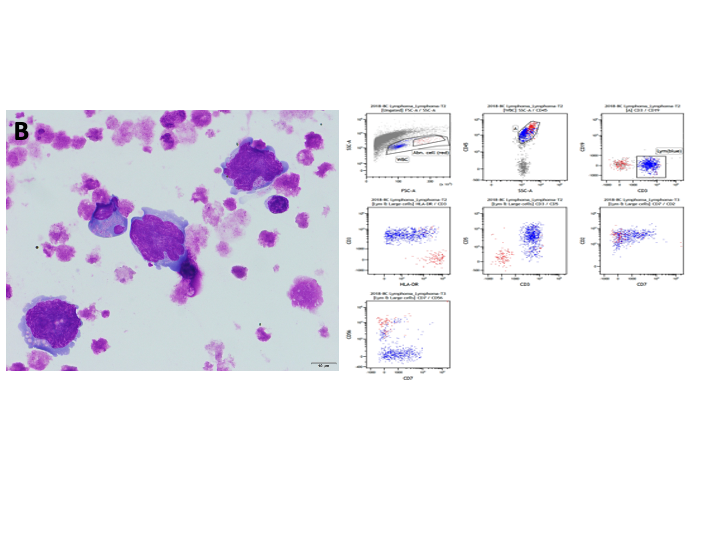 |
| 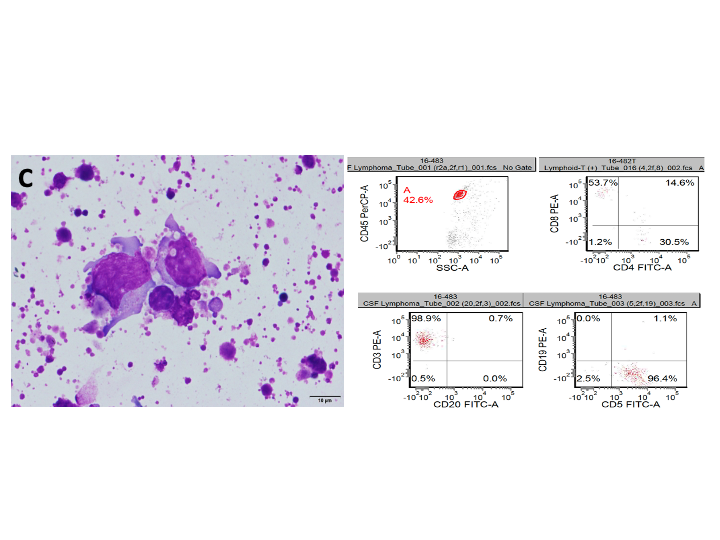 |

Figure. S2

| 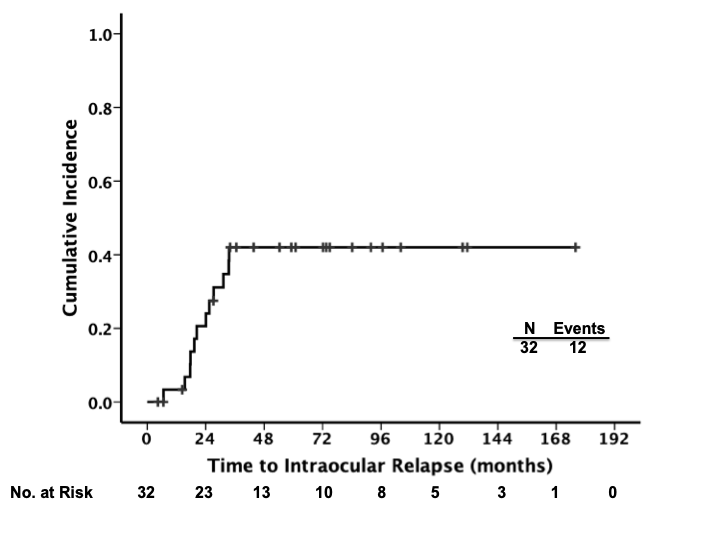 |
| --- |

Figure. S3

| 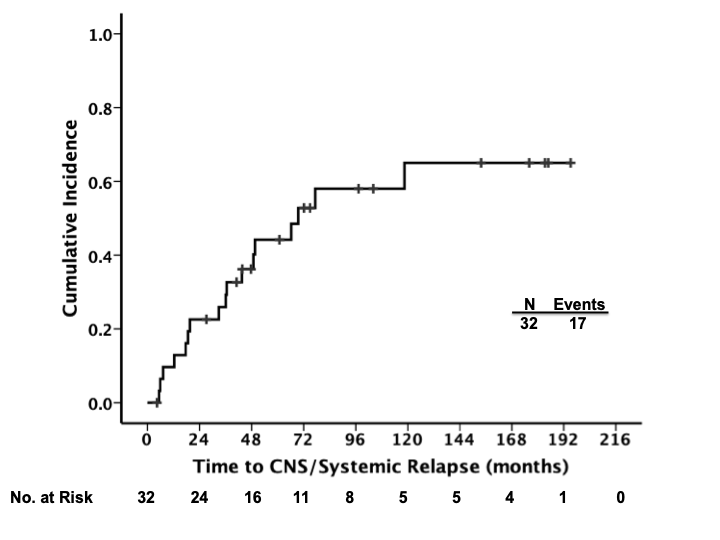 |
| --- |

Figure. S4

| 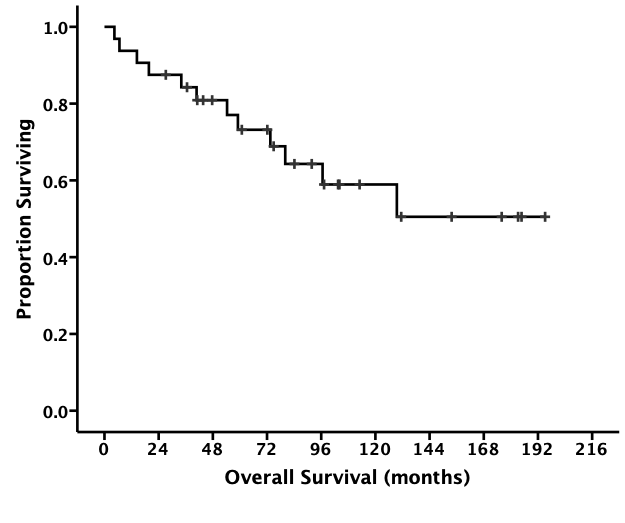  **A**  **No. at Risk 32 28 21 18 12 7 5 4 1 0**  **Median**  **N Events (Months)**  **32 12 Not reached** |
| --- |
| 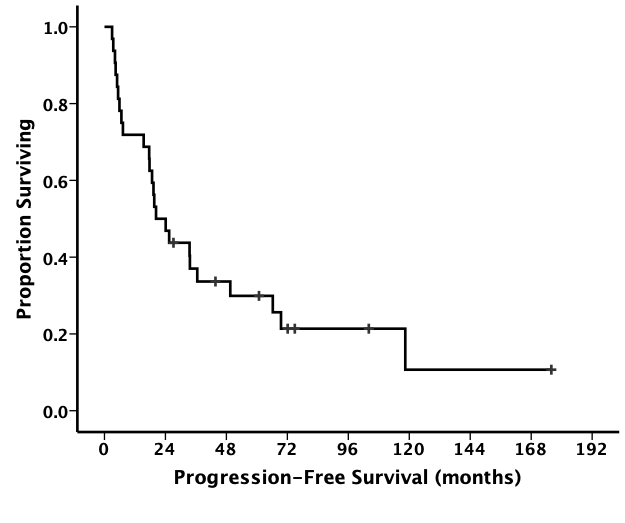  **B**  **No. at Risk 32 16 9 5 3 2 1 1 0**  **Median**  **N Events (Months)**  **32 25 20.3** |

Figure. S5

| 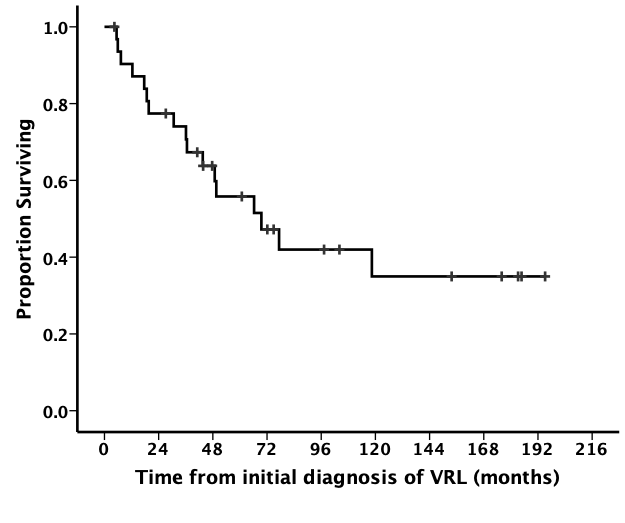  **Median**  **N Events (Months)**  **32 17 69.5**  **No. at Risk 32 24 16 11 8 5 5 4 1 0** |
| --- |

Figure. S6

| 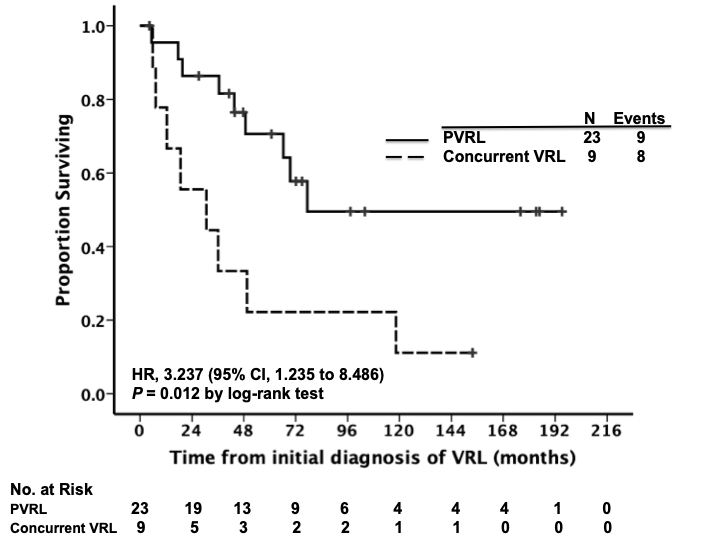 |
| --- |
