# Supplementary material for: Long‐term outcomes of combined intravitreal methotrexate and systemic high‐dose methotrexate therapy in vitreoretinal lymphoma
Source: Cancer Med. 2023 Jan 5;12(7):8102–11. doi: 10.1002/cam4.5609 (PMC10134364; doi:10.1002/cam4.5609)
Supplement: Supplementary file 2 — Appendix S2 [file CAM4-12-8102-s002.docx]

Supplemental Table S1. Treatments and outcomes of the 11 patients with isolated intraocular involvement at the first relapse

|  |  |  | **First relapse** | | | **Second relapse** | | | **Third relapse** | | | **Fourth relapse** | | |
| --- | --- | --- | --- | --- | --- | --- | --- | --- | --- | --- | --- | --- | --- | --- |
| **Case** | **Age at relapse** | **Sex** | **Site of relapse** | **Therapy** | **Outcome** | **Site of relapse** | **Therapy** | **Outcome** | **Site of relapse** | **Therapy** | **Outcome** | **Site of relapse** | **Therapy** | **Outcome** |
| 4 | 75 | M | Bilateral eyes | IVT chemotherapy | CR in 11 m | Bilateral eyes | HDMTX + IVT chemotherapy | CR in 7 m | Bilateral eyes | IVT chemotherapy | CR in 11.5 y |  |  |  |
| 5 | 47 | F | Bilateral eyes | IVT chemotherapy | CR in 11 m | Bilateral eyes | BOMES/BMS + WBRT | CR in 11.7 y |  |  |  |  |  |  |
| 7^#^ | 75 | F | Bilateral eyes | IVT chemotherapy | CR in 11.8 y |  |  |  |  |  |  |  |  |  |
| 8 | 67 | F | Left eye | IVT chemotherapy | CR in 12.4 y |  |  |  |  |  |  |  |  |  |
| 14^#^ | 40 | F | Left eye | IVT chemotherapy | CR in 4.5 m | CNS | BAS + WBRT, BR | Died in 10 m |  |  |  |  |  |  |
| 18 | 55 | F | Left eye | IVT chemotherapy | CR in 17 m | Extensively systemic + CNS | R-CHOP, R-CYVE + ASCT | CR in 60 m |  |  |  |  |  |  |
| 19 | 75 | M | Left eye | IVT chemotherapy | CR in 24.5 m | Right eye | IVT chemotherapy | CR in 7 m | Right eye | IVT chemotherapy | CR in 4.5 m | Lymph nodes | R-miniCHOP | Died in 2.5 m |
| 20 | 79 | F | Left eye | IVT chemotherapy | CR in 20 m | CNS | R-HDMTX + WBRT | CR in 46 m |  |  |  |  |  |  |
| 21 | 56 | F | Bilateral eyes | IVT chemotherapy | CR in 7 m | Bilateral eyes | IVT chemotherapy |  | Bilateral eyes | IVT chemotherapy | CR in 7 m | Bilateral eyes | IVT chemotherapy | CR in 20 m |
| 29 | 53 | M | Bilateral eyes | IVT chemotherapy | CR in 12 m |  |  |  |  |  |  |  |  |  |
| 30 | 71 | F | Right eye | IVT chemotherapy + WBRT | CR in 31.5 m |  |  |  |  |  |  |  |  |  |

^#^Patients with concurrent vitreoretinal lymphoma at diagnosis.

Abbreviations: M, male; F, female; IVT, intravitreal; CR; complete response, HDMTX, high-dose methotrexate; BOMES, carmustine, oncovin, methotrexate, etoposide, and steroid; BMS, carmustine, methotrexate, and steroid; WBRT, whole-brain radiotherapy; CNS, central nervous system; BAS, carmustine, cytarabine, and steroid; BR, bendamustine and rituximab; R, rituximab; CHOP, cyclophosphamide, doxorubicin, oncovin, and predisolone; ASCT, autologous stem cell transplantation; CYVE, cytarabine and etoposide.
